# A Case Study of Dynamic Response Analysis and Safety Assessment for a Suspended Monorail System

**DOI:** 10.3390/ijerph13111121

**Published:** 2016-11-10

**Authors:** Yulong Bao, Yongle Li, Jiajie Ding

**Affiliations:** Department of Bridge Engineering, Southwest Jiaotong University, Chengdu 610031, Sichuan, China; baoyulong1991@163.com (Y.B.); dingjiajieswjtu@163.com (J.D.)

**Keywords:** traffic safety and response, suspended monorail transit system, finite element method, multi-body system dynamics, vehicle-bridge coupling vibration

## Abstract

A suspended monorail transit system is a category of urban rail transit, which is effective in alleviating traffic pressure and injury prevention. Meanwhile, with the advantages of low cost and short construction time, suspended monorail transit systems show vast potential for future development. However, the suspended monorail has not been systematically studied in China, and there is a lack of relevant knowledge and analytical methods. To ensure the health and reliability of a suspended monorail transit system, the driving safety of vehicles and structure dynamic behaviors when vehicles are running on the bridge should be analyzed and evaluated. Based on the method of vehicle-bridge coupling vibration theory, the finite element method (FEM) software ANSYS and multi-body dynamics software SIMPACK are adopted respectively to establish the finite element model for bridge and the multi-body vehicle. A co-simulation method is employed to investigate the vehicle-bridge coupling vibration for the transit system. The traffic operation factors, including train formation, track irregularity and tire stiffness, are incorporated into the models separately to analyze the bridge and vehicle responses. The results show that the coupling of dynamic effects of the suspended monorail system between vehicle and bridge are significant in the case studied, and it is strongly suggested to take necessary measures for vibration suppression. The simulation of track irregularity is a critical factor for its vibration safety, and the track irregularity of A-level road roughness negatively influences the system vibration safety.

## 1. Introduction

With the recent fast expansion of modern cities in China, many types of the urban rail transit system [1,2] have been employed to solve the serious problem of traffic congestion and crashes [3,4]. The suspended monorail transit system, as a kind of urban rail transit system, is an active technological solution. This system has a separate right of way, which has no influence on the current ground transportation system. In the case of crowed urban space and traffic congestion on the road system, it can effectively alleviate traffic pressure and contribute to injury prevention [5,6,7,8,9,10]. Furthermore, suspended monorail transit systems have many advantages, including low manufacturing cost, short construction time and high driving safety and quality, which attracts much attention.

A suspended monorail transit system belongs to a category of urban monorail transit. The monorail vehicle, suspended under the track beam, travels along the rail through the bogie frames located above the vehicle body. Currently, suspended monorail transit systems have been widely developed in Japan and Germany, and a few lines have been operated maturely [11]. As a special type of rail transit, unlike ordinary wheel-rail vehicles, a suspended monorail vehicle employs rubber tires. To ensure the health and reliability of a suspended monorail transit system, it is necessary to study the driving safety of vehicles, and the structure dynamic behavior should also be evaluated [12,13,14]. When suspended monorail vehicles are running on a bridge, the bridge will generate vibration deformation which will enhance the vibration of vehicles, while excessive vibration will make passengers feel uncomfortable and even affect system safety [15,16,17]. The method of coupled vehicle-bridge vibration [18] is usually applied to analyze the dynamic responses of bridge and vehicle, while there is less investigation of vehicle-bridge coupling vibration for the system at present [19].

This paper is focused on establishing a vehicle-bridge coupling vibration model for a suspended monorail transit system with a co-simulation method. An analytical model for the dynamics of the vehicle-bridge system is presented in the time domain in this study with the suspended monorail vehicle and bridge taken as a coupled vibration system [20]. The general FEM software ANSYS and multi-body software SIMPACK [21] are adopted, respectively, to establish the finite element model for the bridge and the multi-body vehicle model; and the co-simulation method of correlating ANSYS and SIMPACK is adopted to investigate the vehicle-bridge coupling vibration. Therefore, the dynamic effect of bridge and vehicle for a suspended monorail system under routine traffic conditions has been studied [22,23]. Comprehensive coverage of all major factors including train formation and track irregularity will be appropriately considered. Additionally, tire stiffness is chosen as a critical factor for driving safety. A bridge model of 25 m-span simple beam is established and selected as a numerical example to study the dynamic performance for suspended monorail transit system.

## 2. Suspended Monorail Bridge Structure

**Figure 1:**
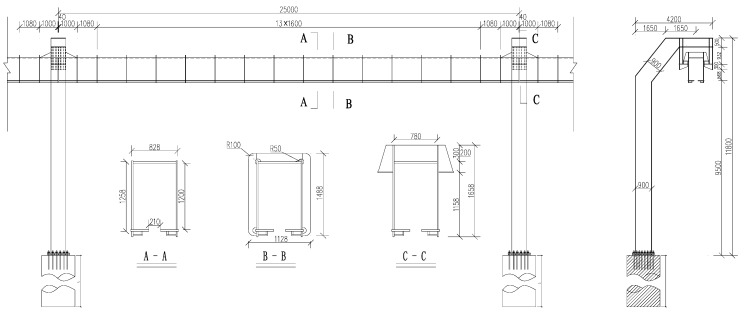
Example bridge structure for this study, in mm.

A suspended monorail structure [24] consists of track beams and columns, similar to the beam and pier in a bridge structure, referred to as suspended monorail bridge structure in this study. A simply-supported bridge structure for the dynamic analysis of suspended monorail transit is shown in Figure 1. It has a main span of 25 m, designed with steel track beams, steel piers and elastomeric pad bearings. All steel members of the bridge are welded steel plate box sections with maximum plate thickness equal to 26 mm. The overall dimensions for the track beam are shown in Figure 1. The analytical model of the bridge is established using the finite element method (FEM). The equation of motion of the whole bridge can be written as:

(1) $$M_{b}{\ddot{u}}_{b}+C_{b}{\dot{u}}_{b}+K_{b}u_{b}=F_{vb},$$

where ${\ddot{u}}_{b}$, ${\dot{u}}_{b}$, $u_{b}$ are, respectively, the vectors of bridge acceleration, velocity and displacement; $M_{b}$, $C_{b}$, $K_{b}$ are the mass matrix, damping matrix and stiffness matrix of the bridge, respectively; $F_{vb}$ are the wheel-rail interactions which are exerted on the running surface by vehicles through wheel-rail interactions.

**Figure 2:**
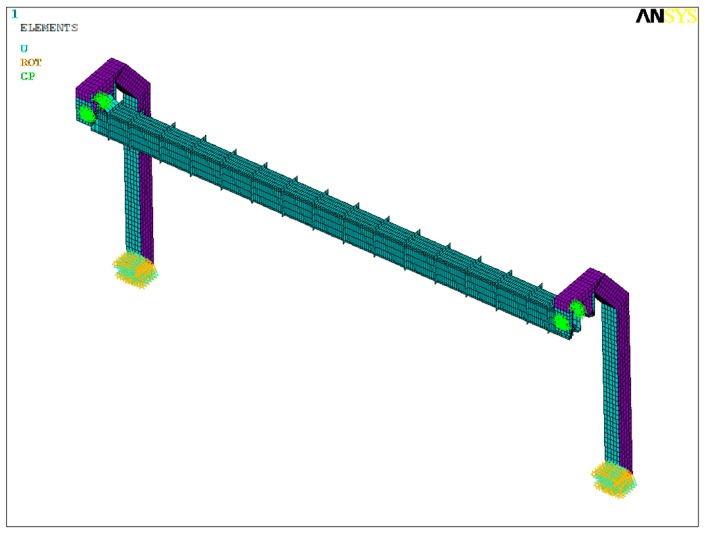
Finite element model of bridge structure.

The software ANSYS is employed to establish the finite element model for the bridge structure. A shell element, Shell63, is applied to model the bridge structure, and the finite element model is shown in Figure 2. The model was auto-meshed, and the minimum grid size of base size element is 80 mm. The entire model was divided into a total of 6489 nodes and 6202 elements. The connection enclosure between girder and pier is considered as a simply-supported system. At the bottom of piers, constraints are fixed at the bottom nodes in the model. The results of natural vibration characteristics for the 25 m-span suspended monorail bridge structure in Table 1 show that the fundamental frequencies of transverse and vertical bending are 1.900 Hz and 6.425 Hz, respectively.

## 3. Modeling of Coupled Vehicle-Bridge System

In order to investigate the dynamic behavior of the bridge structure and the running safety of vehicles for the suspended monorail transit system, a model of vehicle-bridge coupled vibration has to be established first.

### 3.1. Modeling of a Suspended Monorail Vehicle

**Figure 3:**
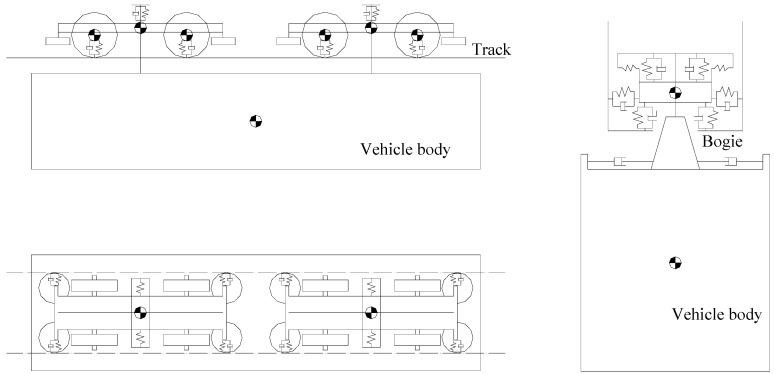
Dynamic model of suspended monorail vehicle.

A suspended monorail train consists of vehicle bodies, wheels, bogies and suspension system, and it can be modeled into a mass-spring-damper system as shown in Figure 3. Differing from a traditional railway vehicle, the bogies of a suspended monorail train are above the vehicle body and the wheels use solid rubber tires. The three-dimensional modeling software CATIA is employed to establish the shape of the suspended monorail transit.

**Figure 4:**
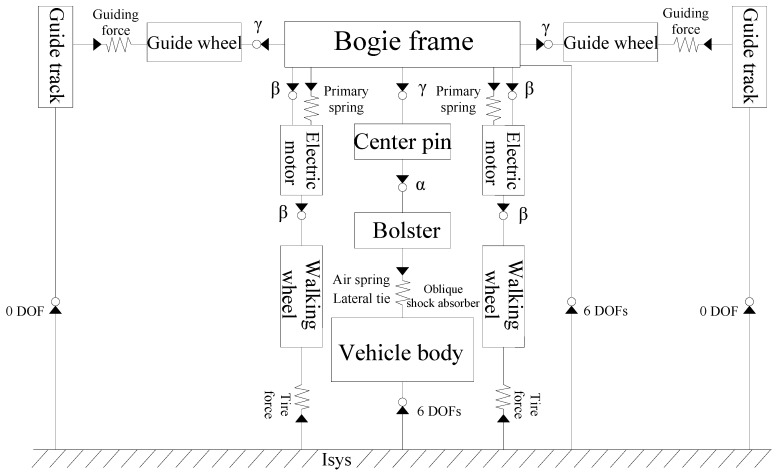
Topological structure of suspended monorail vehicle.

The multi-body dynamics software SIMPACK [21] is adopted to establish a multi-body vehicle model. A suspended monorail train consists of one vehicle body and two bogies, and it can be modeled by a rigid body, a force element and a wheel-rail contact model. The mass of the vehicle body is 10,500 kg, and the major parameters of the suspension system and tires are shown in Table 2. In order to express the relationship and relative movement between the rigid body and inertial coordinate system intuitively, topology is applied to describe the multi-body dynamic structure as shown in Figure 4. A good topology will provide great convenience for the modeling of vehicle dynamics and improve the accuracy of the vehicle model. In this study, the degree of freedom (DOF) along the traveling direction is assumed to be neglected. Thus, each vehicle body and bogies are specified with five degree of freedoms (DOFs), respectively representing vertical, lateral, rolling, yawing and nodding motions. The DOFs of other rigid bodies such as the bolster, center pin and electric motor also have been simplified. Finally, a suspended monorail vehicle with total 43 DOFs is established. The DOFs of each part are described in Table 3.

### 3.2. Track Irregularity

Under the actual operation of a suspended monorail vehicle, due to the fabrication and installation of steel plate for the bridge’s walking surface, walking errors are often unavoidable. For the vehicle-bridge coupled dynamic analysis, the track irregularity is the main mode of excitation of vehicle vibration. However, the wheel-rail contact relation for a suspended monorail transit system is different from a traditional railway vehicle-bridge vibration model [25,26], as the walk wheels of a suspended monorail vehicle use solid rubber tires.

The measurement data of track irregularity for the suspended monorail system is currently unavailable due to the scarcity of previous studies. Considering the unfavorable case in actual operation, road roughness is employed to model the irregularity, referred to as track irregularity in this paper. The road roughness is random. For engineering application, power spectrum density function (PSD) [27] is used to quantify the irregularity of the track approximately, which can be represented as a stationary stochastic process. The expressing equation is shown in Equation (2):

(2) $$G_{q}(n)=G_{q}(n_{0}){\left(\frac{n}{n_{n}}\right)}^{-w},$$

where $G_{q}(n)$ is power spectrum density; *n*, $n_{0}$ are, respectively, the space frequency and reference frequency; *w* is frequency index, *w* = 2 in this study.

**Figure 5:**
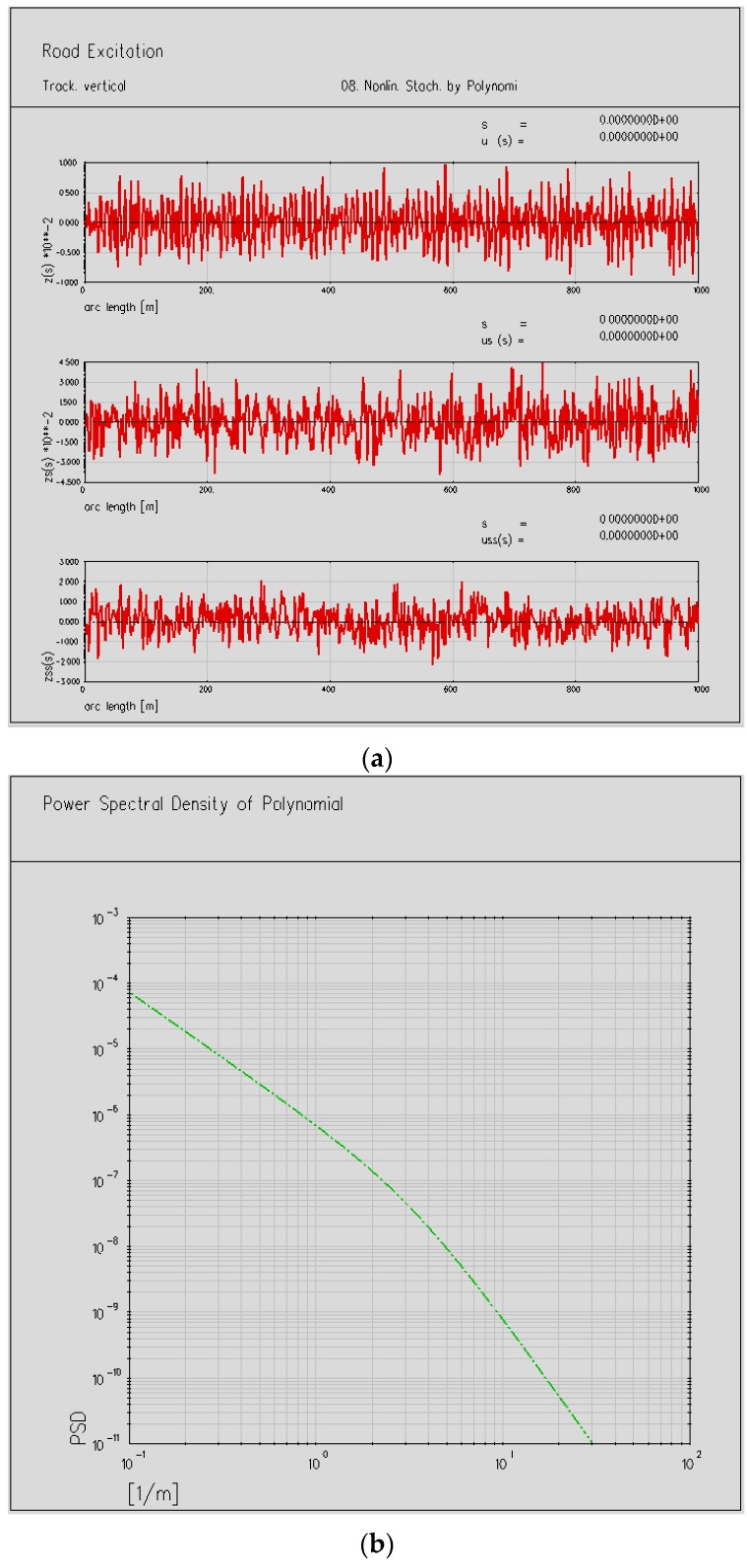
(**a**) The modeling of vertical track irregularities; (**b**) Power spectrum density of vertical track irregularities.

The track irregularities are simulated by the module that comes with software SIMPACK, and taken as system excitation inputs are shown in Figure 5a, and the spectra curves of the model are shown in Figure 5b.

### 3.3. Modeling of Vehicle-Bridge

The dynamic effect of a vehicle–bridge coupled system has been a focus in many recent studies [18,27]. In this study, the existing software SIMPACK is adopted to build the vehicle-bridge structure for the suspended monorail transit system. To study the dynamic responses of the suspended monorail transit system, the co-simulation method is adopted to correlate ANSYS and SIMPACK to investigate the vehicle-bridge coupling vibration model.

The method of substructure analysis is employed to reduce the DOFs of the FEM model, the bridge model is condensed into a single superelement, which contains the matrices of mass, stiffness and modal for the whole bridge model. The substructure technique consists of three steps: generation pass, use pass and expansion pass. The superelement is created in the generation pass, then used in the use pass, and the results are expanded in the expansion pass. In this study, the generation pass and use pass are adopted. The information of mass and stiffness matrices for the bridge structure is condensed into a single superelement and are written into the file of *.sub in the generation pass. The geometry information is also obtained in the file of *.cdb. Modal analysis of the superelement is conducted in the use pass to obtain the modal file of *.rst. After substructure analysis, the information about mass, stiffness, geometry and modes of the bridge structure is saved in the three files, which can be inputted into the SIMPACK.

Guyan condensation method is used in the substructure analysis to reduce the degrees of freedom. The DOFs of the FEM model are divided into master DOFs *u_m_* and auxiliary DOFs *u_s_*. Auxiliary DOFs adhere to master DOFs, and the static equation can be written as:

(3) $$\left[\begin{array}{cc} k_{mm} & k_{ms}\\ k_{sm} & k_{ss} \end{array}\right]\left\{\begin{array}{c} u_{m}\\ u_{s} \end{array}\right\}=\left\{\begin{array}{c} F_{m}\\ F_{s} \end{array}\right\},$$

where *k_mm_* is the stiffness matrix of master DOFs; *k_ss_* is the stiffness matrix of auxiliary DOFs.

**Figure 6:**
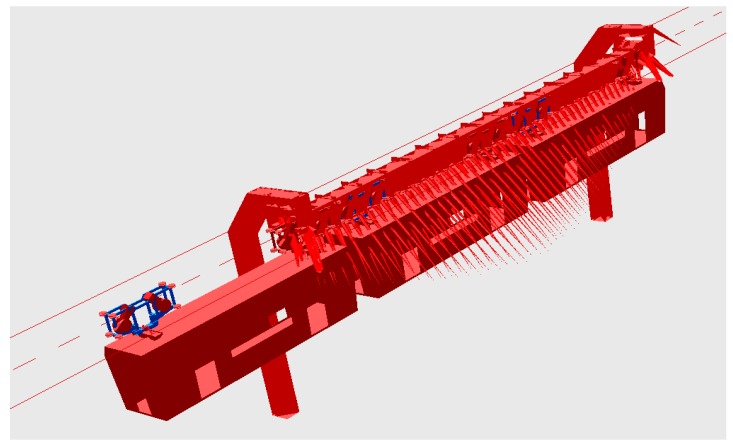
Schematic of vehicle passing through a bridge.

Here is the procedure to realize the co-simulation of ANSYS and SIMPACK. First, substructure analysis should be conducted for the finite element model of a bridge structure in ANSYS to create the essential information files. Then, the bridge model is sent into the multi-body dynamic system with the method of flexible track. Finally, the vehicle-bridge coupling vibration for the suspended monorail transit system is established. Figure 6 shows the response of the dynamic system when the suspended monorail vehicle passing through the bridge.

## 4. Dynamic Analysis of Suspended Monorail Transit System

To study the driving safety of vehicles and dynamic behavior of the bridge structure for suspended monorail transit system [28], a bridge model of 25 m-span simple beam is established and the multi-body dynamic software SIMPACK is employed to obtain the dynamic effects.

### 4.1. Operating Conditions

In order to evaluate the dynamic impact both of suspended monorail vehicle and bridge through vehicle-bridge interactions, the normal operating traffic conditions have to be investigated comprehensively. Under this condition, the train consists of three carriage bodies, running on the bridge at the operating speed of 50 km/h. Track irregularity is not considered for the moment.

#### 4.1.1. Vehicle Dynamic Response

**Figure 7:**
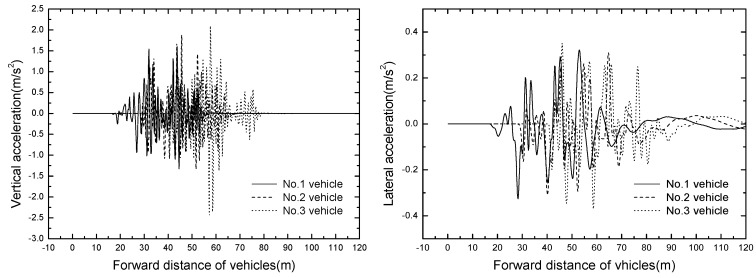
Time histories of vehicle accelerations in operating condition.

The vehicles are numbered from 1 to 3 with the position of vehicles: the head vehicle number is one and the back vehicle number is three. The vehicle responses at the speed of 50 km/h are shown in Table 4. The time histories of vertical and lateral acceleration of vehicles are displayed in Figure 7.

It can be seen from Table 3 that the maximum vertical and lateral acceleration are 2.433 m/s^2^ and 0.370 m/s^2^ respectively, the vertical acceleration exceeds the limit of 2.0 m/s^2^. Thus, it is necessary to optimize the primary spring and tire stiffness to reduce the vertical vibration of the vehicle. The value of Sperling index is less than 2.5, showing that the general comfortable index is excellent. Because this study only considers the suspended monorail vehicle travelling along a straight line, the slip angle of vehicle body is very small, far from meeting the requirement of running safety.

As shown in Figure 7, No. 1 vehicle is the first to enter and leave the bridge, and the vertical acceleration responses increase with the increases of vehicle number. The result shows that the front vehicle amplifies the bridge vertical displacement, which expands the vertical responses of vehicles entering the bridge later.

#### 4.1.2. Bridge Dynamic Response

**Figure 8:**
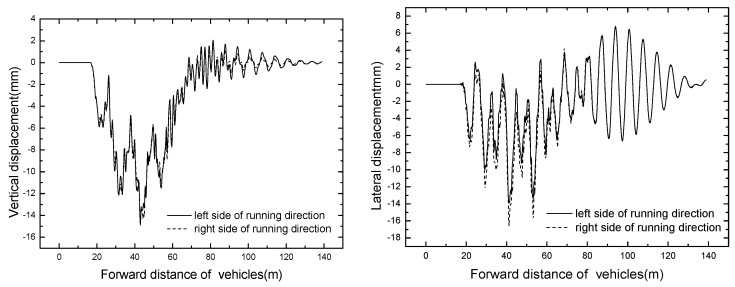
Time histories of displacement of mid span.

**Figure 9:**
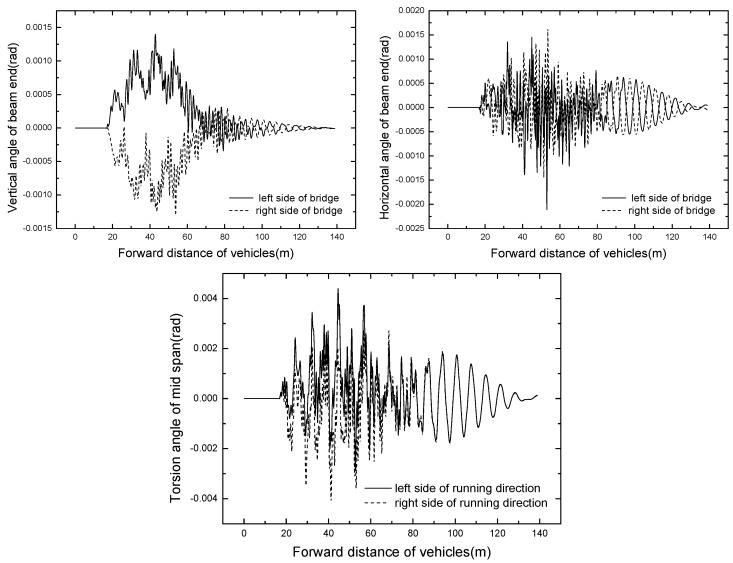
Time histories of rotation and torsion angles.

**Figure 10:**
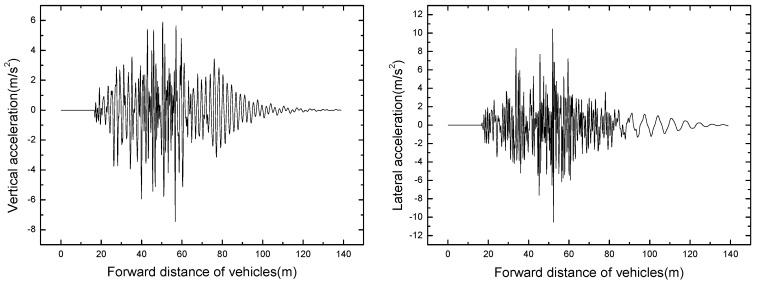
Time histories of accelerations of mid span in operating condition.

In this study, the bridge structure is modelled precisely by shell elements, so there exists two symmetrical contact points under the walking wheels at the mid span: left and right bridge contact points. Without special notes, bridge contact point under the left side walking wheel is employed to show the time histories of bridge responses. The time histories of displacement and acceleration at mid span are displayed in Figure 8, Figure 9 and Figure 10, respectively.

As shown in Figure 8 and Figure 9, the curves of time histories for bridge displacement meet the general rule, and the bridge responses of both left and right bridge contact points are basically identical. Because the transverse stiffness of the pier structure is smaller, the global lateral displacement of the mid span is not symmetric. It can be seen from Figure 10 that the vertical and lateral accelerations of the mid span are large. Because the basic lateral bending frequency is lower, close to the lateral frequency of the vehicle-bridge system, this leads to the lateral acceleration of mid span being larger than the vertical acceleration. Meanwhile, the bridge points are in direct contact with walking wheels, and the damping effect of the steel bridge is poor, thus the acceleration responses of the bridge are large. Effective measures have to be taken to reduce the bridge vibration, for instance, a vibration cushion or shock absorber should be assigned and tire stiffness should be reduced appropriately to ensure the bridge safety.

### 4.2. The Influence of Train Formation

The sensitivity analysis of loading conditions is conducted in this study. The train formation plans of one vehicle, two vehicles and three vehicles are employed respectively as loading conditions. The vehicle speed is 50 km/h, track irregularity is not considered in this case.

#### 4.2.1. Vehicle Dynamic Response

The vehicle responses of different train formation conditions are shown in Table 5. It can be seen that the No. 3 vehicle of three vehicles has the maximum vertical and lateral accelerations, which approach 2.433 m/s^2^ and 0.370 m/s^2^ respectively.

#### 4.2.2. Bridge Dynamic Response

**Figure 11:**
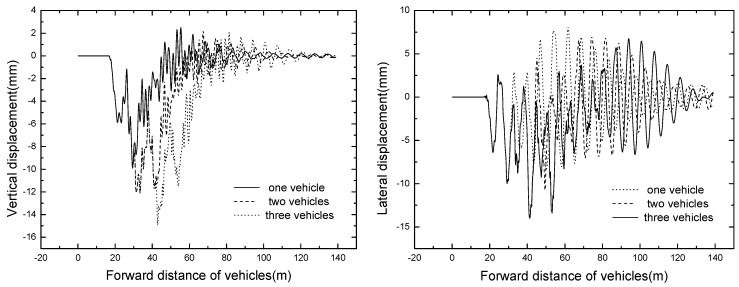
Time histories for displacement of mid span of different train formations.

**Figure 12:**
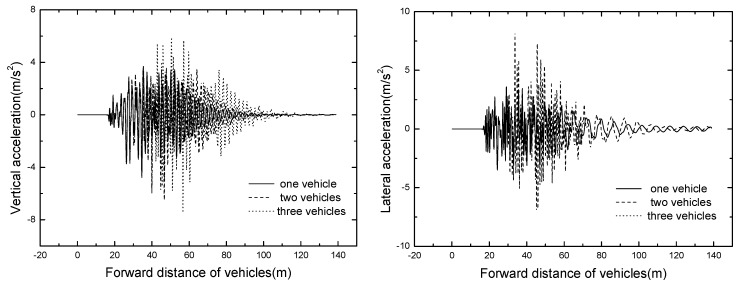
Time histories for acceleration of mid span of different train formations.

The bridge responses of different train formation conditions are shown in Table 6. The time histories of displacement and acceleration of mid span with different train formation conditions are displayed in Figure 11 and Figure 12.

It can be seen that the bridge responses increase with the increase of train formation. When three vehicles are passing through the bridge, the vertical displacement and acceleration reach the maximum value of 14.90 mm and 7.46 m/s^2^ respectively.

### 4.3. The Influence of Track Irregularity

Track irregularity is the main source of excitation for a vehicle-bridge coupled dynamic system. In order to study the influence of track irregularity, the national A-level standard road roughness in China is considered, the modeling of track irregularity is shown in Section 3.1. The vehicle speed is 50 km/h, and the train formation adopts three vehicles, the vehicle-bridge coupled vibration analysis is conducted, and the dynamic responses of vehicle and bridge are calculated.

#### 4.3.1. Vehicle Dynamic Response

**Figure 13:**
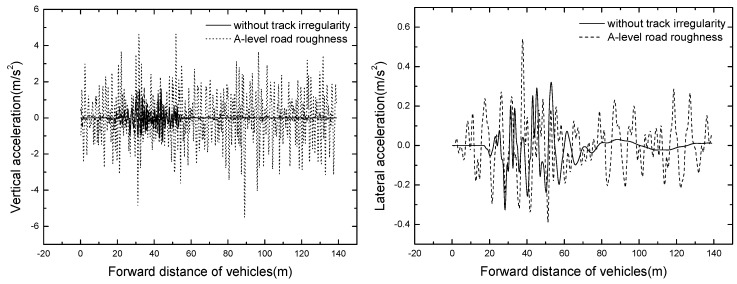
Time histories of No. 1 vehicle accelerations.

The vehicle responses of different track irregularity are shown in Table 7. The time histories of No. 1 vehicle accelerations are displayed in Figure 13.

It can be seen from Table 7 and Figure 13 that the vehicle vertical responses of A-level road roughness are much bigger than that without track irregularity, and the maximum value of vertical acceleration is 5.496 m/s^2^. It can be concluded that the dynamic responses of suspended monorail vehicle are highly sensitivity to the track irregularity, while the walking surface of the suspended monorail is paved with prefabricated steel plate, which has good flatness. Thus, it is inappropriate to adopt A-level road roughness as the track irregularity.

#### 4.3.2. Bridge Dynamic Response

**Figure 14:**
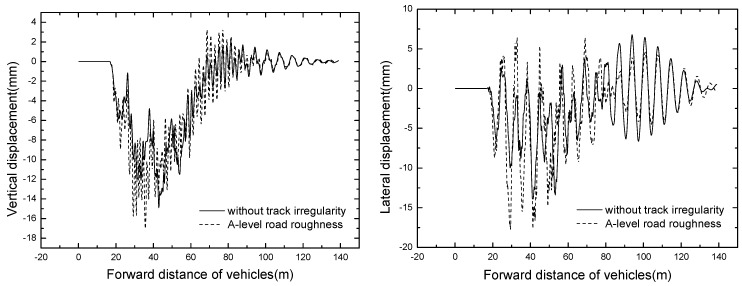
Time histories of displacement of mid span of different track irregularities.

**Figure 15:**
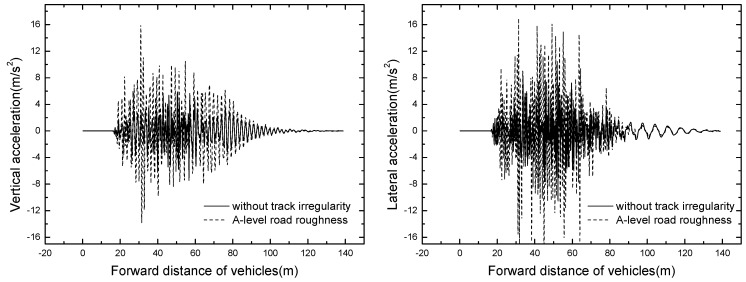
Time histories of acceleration of mid span with different track irregularities.

The bridge responses of different track irregularity are shown in Table 8. The time histories of displacement and acceleration of mid span with different track irregularity are displayed in Figure 14 and Figure 15.

It can be seen in the Figure 14 and Figure 15 that track irregularity has great influence on the dynamic responses of the bridge. The maximum dynamic displacement and acceleration all come from the track irregularity of A-level road roughness. Hence, it has negative influence on the dynamic responses of both vehicles and bridges with A-level road roughness range.

## 5. Sensitivity Analysis of Tire Stiffness

In this study, the suspended monorail vehicle uses solid rubber tires, which applies to the condition of low operating speed and high load, and it has the advantage of long cycle life and wear-resistance. Meanwhile, the solid rubber tire has great stiffness, which leads to the poor adaptability to road roughness. Thus, it is necessary to conduct the sensitivity analysis of tire stiffness.

In order to study the influence of tire stiffness to the vehicle-bridge coupled system, the tire stiffnesses of 1.0 × 10^6^ N/m, 2.0 × 10^6^ N/m and 4.0 × 10^6^ N/m are adopted, respectively, in which 4.0 × 10^6^ N/m is the actual stiffness of a solid rubber tire for suspended monorail vehicles. The vehicle speed is 50 km/h, the train formation adopts three vehicles, and track irregularity is not considered for the moment. The analysis of vehicle-bridge coupled vibration is conducted, and the dynamic responses of vehicle and bridge are calculated.

### 5.1. Vehicle Dynamic Response

**Figure 16:**
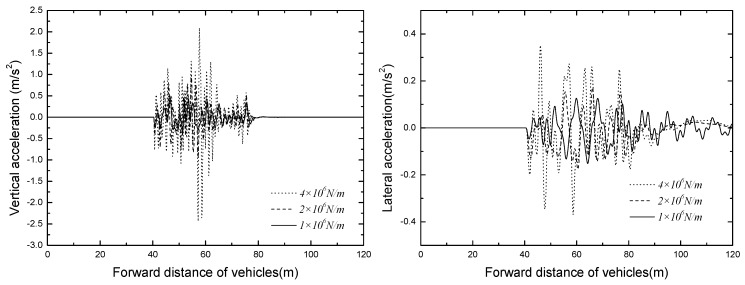
Time histories for No. 3 vehicle accelerations.

The time histories of No. 3 vehicle accelerations of different tire stiffness are displayed in Figure 16. The vehicle responses with different tire stiffness are shown in Table 9.

It can be seen in the Figure 16 and Table 9 that the maximum value of vehicle acceleration appears in the condition with tire stiffness is 4.0 × 10^6^ N/m, the vertical and lateral accelerations are 2.433 m/s^2^ and 0.370 m/s^2^ respectively. Nevertheless, the slip angle of vehicle body decreases with the increase of tire stiffness, when the tire stiffness value is 1.0 × 10^6^ N/m, the slip angle approaches the maximum value of 0.0179 rad. Overall, when the suspended monorail vehicle uses solid rubber tires, due to the greater stiffness, it has poorer adaptability to roughness.

### 5.2. Bridge Dynamic Response

**Figure 17:**
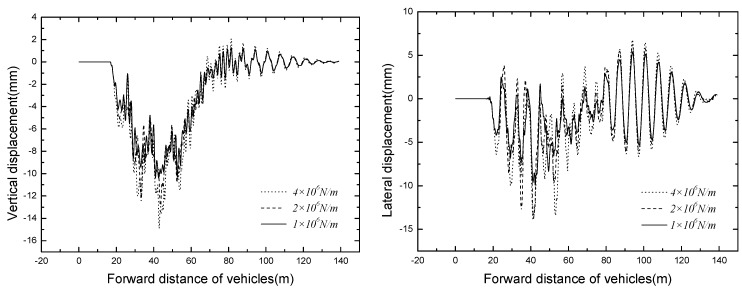
Time histories for displacement of mid span of different tire stiffnesses.

**Figure 18:**
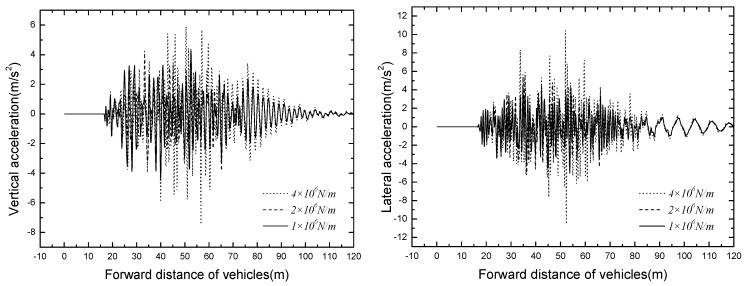
Time histories for acceleration of mid span of different tire stiffnesses.

The time histories for displacement and acceleration of mid span of different tire stiffness are displayed in Figure 17 and Figure 18. The results of tire stiffness sensitivity analysis of bridge responses are shown in Table 10.

As shown in Figure 17 and Figure 18, the bridge responses decrease significantly with the decrease of tire stiffness, the vertical and lateral dynamic displacements of mid span can even decrease 4.5 mm and 4.0 mm, respectively. The mid span accelerations also decrease with the decrease of tire stiffness, but when the value of tire stiffness is decreased to a certain extent, the responses of bridge acceleration are no longer under its influence. Thus, an appropriate tire stiffness should be adopted to ensure the operating safety of suspended monorail vehicles.

## 6. Conclusions

In order to evaluate the safety and reliability of suspended monorail transit system, a detailed numerical study was conducted to investigate the transit driving safety of vehicles and the dynamic behavior of bridge structure. An analytical model of vehicle-bridge coupled vibration for suspended monorail system is established, and several influent factors are studied to ensure the health of system. Some interesting conclusions can be obtained through this study, including:

1. A vehicle-bridge coupling vibration model for the suspended monorail transit system with a co-simulation method is established. The coupling dynamic effects of suspended monorail system between vehicle and bridge are significant in the case studied. It is necessary to study the impact effects of suspended monorail system based on the method of coupled vehicle-bridge vibration.
2. The suspended monorail vehicles cause larger bridge acceleration responses, which exceed the dynamic effect allowance of the traditional railway bridge evaluation criteria. It is strongly suggested to take necessary measures for vibration suppression.
3. Comparing to short train formation, the dynamic responses of the bridge increase significantly in longer train formation condition.
4. Track irregularity will have a significant negative influence on the dynamic responses of both vehicles and bridges. The walking surface for suspended monorail vehicle has good flatness, so it might be inappropriate to adopt A-level road roughness as the track irregularity. The simulation of track irregularity is a critical factor for its vibration safety, and analysis including more track irregularity types should be studied in the future.
5. The dynamic responses of suspended monorail systems are sensitive to the tire stiffness. The study shows that with a slight decrease of the tire stiffness, the dynamic responses are weakened significantly.

## Figures and Tables

**Table 1:** Frequency and vibration shape of the bridge.

Order	Frequency/Hz	Mode Shape
1	1.900	Track beam lateral symmetric bending-1
2	2.133	Track beam longitudinal drifting
3	3.559	Track beam lateral antisymmetric bending-1
4	3.940	Track beam lateral symmetric bending-2
5	4.447	Pier longitudinal drifting
6	6.425	Track beam vertical symmetric bending-1
7	8.351	Track beam lateral antisymmetric bending-2
8	11.638	Track beam vertical symmetric bending-2
9	13.346	Track beam lateral antisymmetric bending-3
10	13.801	Track beam lateral symmetric bending-3

**Table 2:** Major parameters of the suspension system and tires.

Members	Stiffness (N/m)			Vertical Damping (Ns/m)
	Vertical	Transverse	Longitudinal	
Air spring	0.3 × 10^6^	0.1 × 10^6^	0.1 × 10^6^	25,000
Primary spring	1.5 × 10^6^	——	——	15,000
**Members**	**Radial Stiffness (N/m)**			**Radial Damping (Ns/m)**
Walking wheel	4.0 × 10^6^			1000
Guide wheel	0.8 × 10^6^			3000

**Table 3:** DOFs of dynamic model of suspended monorail vehicle.

DOFs	Lateral	Vetical	Rolling	Yawing	Nodding
Verhicle body	Y_c_	Z_c_	φ_c_	ψ_c_	θ_c_
Bogie (i = 1, 2)	Y_ti_	Z_ti_	φ_ti_	ψ_ti_	θ_ti_
Walking wheel (i = 1–4)	——	——	——	——	θ_zi_
Guide wheel (i = 1–8)	——	——	——	Ψ_di_	——
Bolster (i = 1, 2)	——	——	φ_yi_	——	——
Center pin (i = 1, 2)	——	——	——	Ψ_si_	——
Electric motor (i = 1, 2)	——	——	——	——	θ_xi_

**Table 4:** Vehicle responses in operating condition (v = 50 km/h).

Vehicle Number		No. 1	No. 2	No. 3
Lateral acceleration (m/s^2^)		0.326	0.310	0.370
Vertical acceleration (m/s^2^)		1.598	1.875	2.433
Sperling	Lateral	1.449	1.396	1.531
	Vertical	1.749	1.745	1.834
Slip angle of vehicle (rad/1000)		5.884	6.545	6.516

**Table 5:** Vehicle responses of different train formation.

Train Formation		One Vehicle	Two Vehicles		Three Vehicles		
Vehicle Number		No. 1	No. 1	No. 2	No. 1	No. 2	No. 3
Lateral acceleration (m/s^2^)		0.326	0.326	0.365	0.326	0.310	0.370
Vertical acceleration (m/s^2^)		1.146	1.546	1.554	1.598	1.875	2.433
Sperling	Lateral	1.427	1.441	1.399	1.396	1.531	0.950
	Vertical	1.674	1.706	1.695	1.745	1.834	1.954

**Table 6:** Maximum values of bridge responses of different train formation.

Train Formation	Dynamic Displacement (mm)		Acceleration (m/s^2^)		Torsion Angle (Rad/1000)	Vertical Angle of Beam End (Rad/1000)		Horizontal Angle of Beam End (Rad/1000)	
	Vertical	Lateral	Vertical	Lateral		Left	Right	Left	Right
One vehicle	9.76	10.03	4.78	3.58	2.74	0.99	1.29	0.85	0.93
Two vehicles	12.08	14.12	6.49	8.21	3.88	1.16	1.12	1.82	1.18
There vehicles	14.90	13.90	7.46	10.54	4.40	1.40	1.30	2.11	1.62

**Table 7:** Vehicle responses of different track irregularity.

Vehicle Number	Track Irregularity	Vertical Acceleration (m/s^2^)	Lateral Acceleration (m/s^2^)	Sperling		Slip Angle of Vehicle (Rad/1000)
				Vertical	Lateral	
No. 1	Without track irregularity	1.598	0.326	1.749	1.449	5.884
	A-level road roughness	5.496	0.539	3.371	1.922	31.530
No. 2	Without track irregularity	1.875	0.310	1.745	1.396	6.545
	A-level road roughness	4.455	0.423	3.373	1.903	42.25
No. 3	Without track irregularity	2.433	0.370	1.834	1.531	6.516
	A-level road roughness	4.767	0.296	3.270	1.634	27.300

**Table 8:** Maximum values of bridge responses of different track irregularity.

Track Irregularity	Dynamic Displacement (mm)		Acceleration (m/s^2^)		Torsion Angle (Rad/1000)	Vertical Angle of Beam End (Rad/1000)		Horizontal Angle of Beam End (Rad/1000)	
	Vertical	Lateral	Vertical	Lateral		Left	Right	Left	Right
Without track irregularity	14.90	13.90	7.46	10.54	4.40	1.40	1.30	2.11	1.62
A-level road roughness	17.02	17.74	15.99	19.30	7.34	1.70	2.23	2.59	2.23

**Table 9:** Vehicle responses of different tire stiffness.

Vehicle Number	Tire Stiffness (N/m)	Vertical Acceleration (m/s^2^)	Lateral Acceleration (m/s^2^)	Sperling		Slip Angle of Vehicle (Rad/1000)
				Vertical	Lateral	
No. 1	1.0 × 10^6^	0.629	0.304	1.447	1.231	16.113
	2.0 × 10^6^	1.750	0.335	1.659	1.500	5.081
	4.0 × 10^6^	1.598	0.326	1.749	1.449	5.884
No. 2	1.0 × 10^6^	0.503	0.257	1.630	1.132	17.943
	2.0 × 10^6^	1.427	0.367	1.748	1.462	5.607
	4.0 × 10^6^	1.875	0.310	1.745	1.396	6.545
No. 3	1.0 × 10^6^	0.498	0.151	1.383	1.290	14.212
	2.0 × 10^6^	0.999	0.176	1.630	1.266	4.703
	4.0 × 10^6^	2.433	0.370	1.834	1.531	6.516

**Table 10:** Maximum values of bridge responses of different tire stiffness.

Tire Stiffness (N/m)	Dynamic Displacement (mm)		Acceleration (m/s^2^)		Torsion Angle (Rad/1000)	Vertical Angle of Beam End (Rad/1000)		Horizontal Angle of Beam End (Rad/1000)	
	Vertical	Lateral	Vertical	Lateral		Left	Right	Left	Right
1.0 × 10^6^	10.4	9.9	4.50	5.03	4.33	1.08	1.08	1.48	1.08
2.0 × 10^6^	13.1	13.7	4.29	5.54	4.37	1.14	1.22	1.45	1.39
4.0 × 10^6^	14.9	13.9	7.46	10.54	4.40	1.40	1.30	2.11	1.62
